# Consensus and contention in beauty judgment

**DOI:** 10.1016/j.isci.2024.110213

**Published:** 2024-06-08

**Authors:** Maria Pombo, Aleksandra Igdalova, Denis G. Pelli

**Affiliations:** 1 Department of Psychology, New York University, New York, NY, USA; 2 Department of Psychology, Goldsmiths, University of London, London, UK; 3 Center for Neural Science, New York University, New York, NY, USA

**Keywords:** Social interaction, Social sciences

## Abstract

Variance across participants is at the heart of the centuries-old debate about the universality of beauty. Beauty's belonging to the eye of the beholder implies large interindividual variance, while beauty as a universal object property implies the opposite. To characterize the variance at the center of this debate, we selected two quartets with either high- or low-variance images with high typicality and a given mean beauty. The quartets have high or low variance across 50 participants (*group* variance) and correspondingly high or low variance across images of a quartet for each participant (*quartet* variance). We asked 52 new participants to estimate their own mean and quartet variance. Participants successfully predicted their quartet mean but failed to predict their quartet variance. Though invisible, beauty variance is essential to prediction, both in theory and in practice. The quartets show that mean beauty is not the whole story — beauty variance is heterogeneous.

## Highlights

• The Disputed- and Undisputed-Beauty Quartets show extreme beauty judgment variance.
• The former's beauty ratings are better fit by 2 Gaussians, and the latter's by 1.
• Participants could predict their quartet mean but not their quartet variance.
• The quartets show that the mean is not enough to understand beauty judgment.

## Graphical abstract

**Figure:**
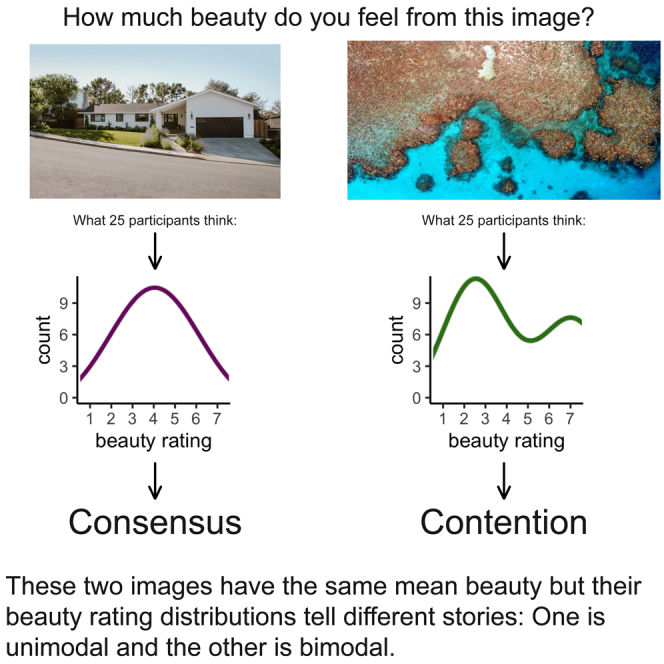

## Introduction

Is beauty a universal object property or in the eye of the beholder? Variance across participants lies at the core of this age-old debate. The former implies consensus, or small interindividual variance, while the latter implies contention, or large interindividual variance.

Empirical aesthetics has yielded evidence supporting both consensus and contention in beauty judgment, and the weight of evidence on this contest has shifted historically. The statistical assessment of contention in subjective judgments originated in the early days of empirical aesthetics.^1^ For example, scholars in the field pointed to the large interindividual differences in the strength of pleasantness reactions^2^^,^^3^ and color preference.^4^ However, the focus on contention was overshadowed by the rise of behaviorism, and later by Berlyne’s^5^ theories relating object properties to hedonic responses. Individual differences re-emerged as an area of focus in empirical aesthetics only in the last decade.^6^

### Consensus

Certain stimulus-based characteristics correlate reliably with aesthetic preference. One well-known example is symmetry,^7^ which has been found to predict both implicit^8^ and explicit preferences for random dot configurations,^9^ even across cultures.^10^ Evidence also suggests a reliable preference for curved contours in images ranging from abstract patterns to real objects.^11^^,^^12^ Furthermore, others find stable preferences for color properties such as hue, lightness, and saturation, as well as for certain spatial compositions, and the golden ratio.^13^

### Contention

Much of the research on aesthetic contention measures how much variance in aesthetic judgment can be attributed to individual or shared preferences.^14^^,^^15^^,^^16^^,^^17^^,^^18^ A common finding is that judgment idiosyncrasy explains at least half the variance in aesthetic judgment. Some have pointed out contention in preference for different stimulus types such as art styles^19^ or movies^20^ and stimulus features such as color, symmetry, and complexity.^21^^,^^22^^,^^23^ Others have tied these individual differences to diversity in expertise or ideology.^24^^,^^25^ Researchers have also identified individual differences in aesthetic sensitivity^26^ and have developed different questionnaires meant to characterize these differences in music and aesthetic experiences in general.^27^^,^^28^^,^^29^ Furthermore, metrics to assess individual differences have been developed. For example, “taste typicality”^30^ measures how likely an individual is to match the mean aesthetic preference of a group, and “evaluation bias”^31^ measures, for a single participant, how consistent their aesthetic judgments are for a given category (e.g., faces). Recently, researchers have also found individual differences in metrics like “aesthetic stability”, i.e., how stable individual preference is over time.^32^

### Current study

**Figure 1:**
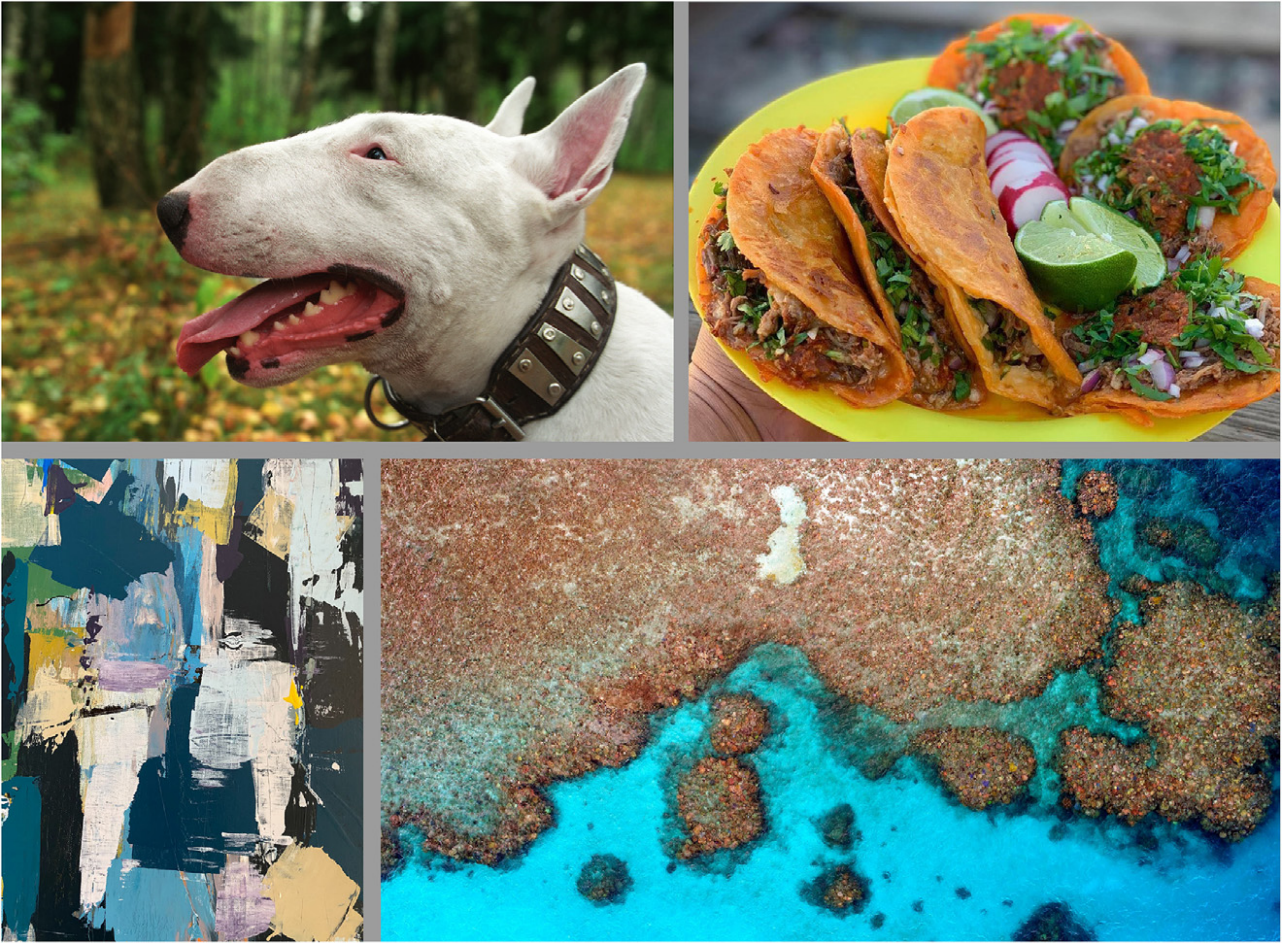
The Disputed-Beauty Quartet (Top Left) Bull Terrier. Obtained from i-Stock.com/ingret. (Top Right) Tacos. Published with permission from @teddysredtacos. (Bottom Left) Abstract Art. Detail from “Sea-Dweller” by Vojtech Bruzek. Acrylic, 150x100cm. Obtained from https://unsplash.com/photos/zMl9PjGFPWg. (Bottom Right) Coral Reef. Photo of Farquharson Reef, Australia by GeoNadir. Obtained from https://unsplash.com/photos/b78E12dTxlo.

**Figure 2:**
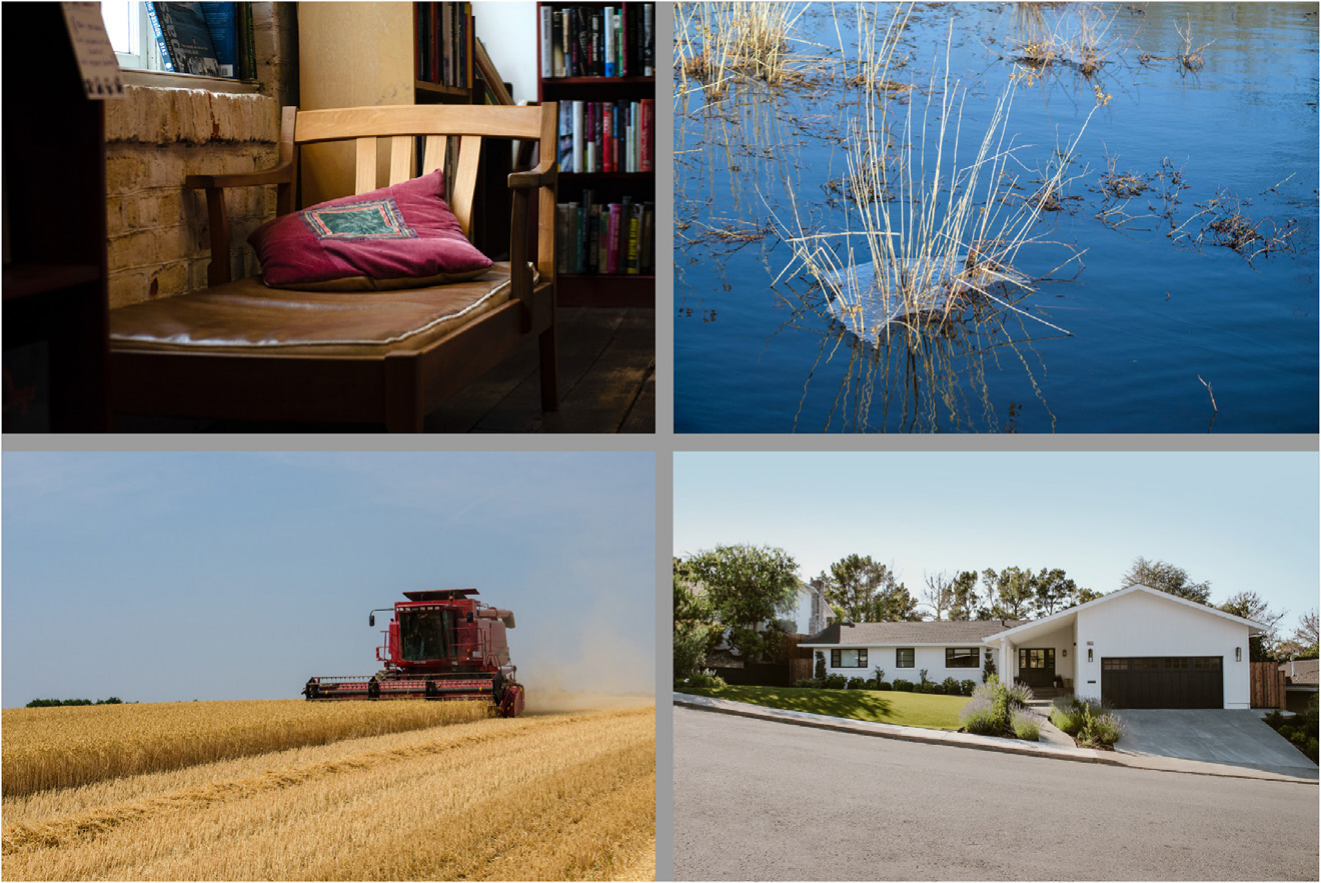
The Undisputed-Beauty Quartet (Top Left) Bookshop. Obtained from https://unsplash.com/photos/47fcqcU1b7k. (Top Right) Reeds. Obtained from https://unsplash.com/photos/BlOBWQ9dkLU. (Bottom Left) Farm. Obtained from https://www.pxfuel.com/en/free-photo-qduou. (Bottom Right) House. Published with permission from Yardzen (@yardzen; https://yardzen.com/).

Here, we want to raise awareness about variance in everyday experiences of beauty. We present two image quartets, the Disputed-Beauty Quartet and the Undisputed-Beauty Quartet (Figures 1 and 2), which exemplify variance at both extremes. The quartets are composed of typical everyday images that have the same mean beauty rating. They have high or low variance across participants (*group* variance) and correspondingly high or low variance across images in a quartet for each participant (*quartet* variance). After finding that members of the lab did not notice the 2-fold difference in variance between the quartets, we replicated the variance measurements of the quartets on another sample and tested how well participants could estimate the quartet variance. Participants completed one of three tasks: an image crowdsourcing task, an image rating task, or a variance estimation task (for full details see STAR Methods).

## Results

### Image crowdsourcing task

To select a set of images, we crowdsourced images with high and low beauty variance. First, we obtained images suggested by lab members, family, and friends. Then online participants completed a simple image crowdsourcing task. We asked them to submit photos or links to photos that were either disputed or undisputed in terms of their beauty. In the end, this resulted in 182 disputed-beauty images and 180 undisputed-beauty images.

### Image rating task

**Figure 3:**
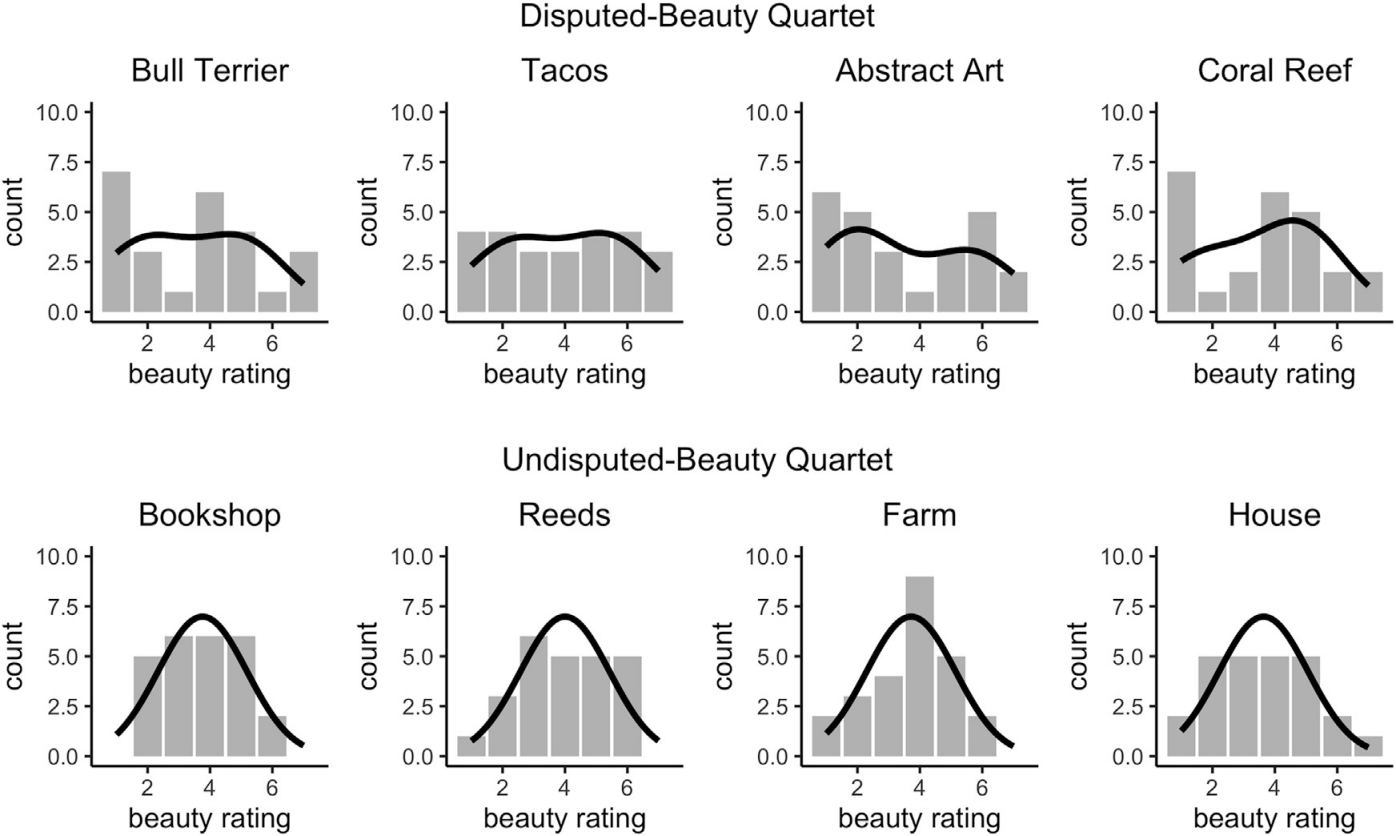
Beauty rating distributions for all images in the quartets The top row corresponds to the images in the Disputed-Beauty Quartet, which peak at low and high numbers (Figure 1; Table 1) and the bottom to the Undisputed-Beauty Quartet, which peak at the center (Figure 2; Table 2). Solid lines correspond to the best model fit.

A new group of participants rated the beauty and typicality of either the disputed- or undisputed-beauty images on a 7-point Likert scale. For each image, we used these ratings to create two quartets. The Disputed-Beauty Quartet (Figure 1) contains four images with high typicality, mean beauty ratings between 3.5 and 4, and beauty standard deviation above 2. The Undisputed-Beauty Quartet (Figure 2) contains four images with high typicality, mean beauty ratings between 3.5 and 4, and beauty standard deviation below 1.6. Tables 1 and 2 contain the quartet image statistics and Figure 3 displays the distributions of beauty ratings.

**Table 1:** Disputed-Beauty Quartet statistics

Image	Beauty Mean	Beauty *SD*	Typicality
Bull Terrier	3.5	2.1	4.8
Tacos	3.9	2.1	5.5
Abstract Art	3.5	2.2	3
Coral Reef	3.6	2	3.8

**Table 2:** Undisputed-Beauty Quartet statistics

Image	Beauty Mean	Beauty *SD*	Typicality
Bookshop	3.8	1.3	3.8
Reeds	4.0	1.5	4.3
Farm	3.7	1.4	4.5
House	3.6	1.6	5.8

For the Disputed-Beauty Quartet, the beauty rating distributions appear multimodal. Beauty ratings peak at low and high numbers. In contrast, the beauty rating distributions of the images in the Undisputed-Beauty Quartet appear normal. In all cases, at most two out of 25 participants rated the beauty of any of the images a 1 or a 7.

As expected, one-sided, two-sample *t*-tests indicate a significant difference between the quartets’ standard deviations, *t*(6) = 8.51, *p* < 0.001, *d* = 6.02, and no significant difference between the quartets' means, *t*(6) = −1.18, *p* = 0.86, *d* = 0.83. Between the Disputed- and Undisputed-Beauty quartets, the mean standard deviation differs by a factor of 1.5 (which corresponds to a factor of 2.25 in variance). In bounded scales like our Likert beauty scale, standard deviation decreases near the ends.^14^ Here, we restricted the quartets to have middle-ranged beauty values (between 3.5 and 4), which limits that range of possible standard deviations.

**Figure 4:**
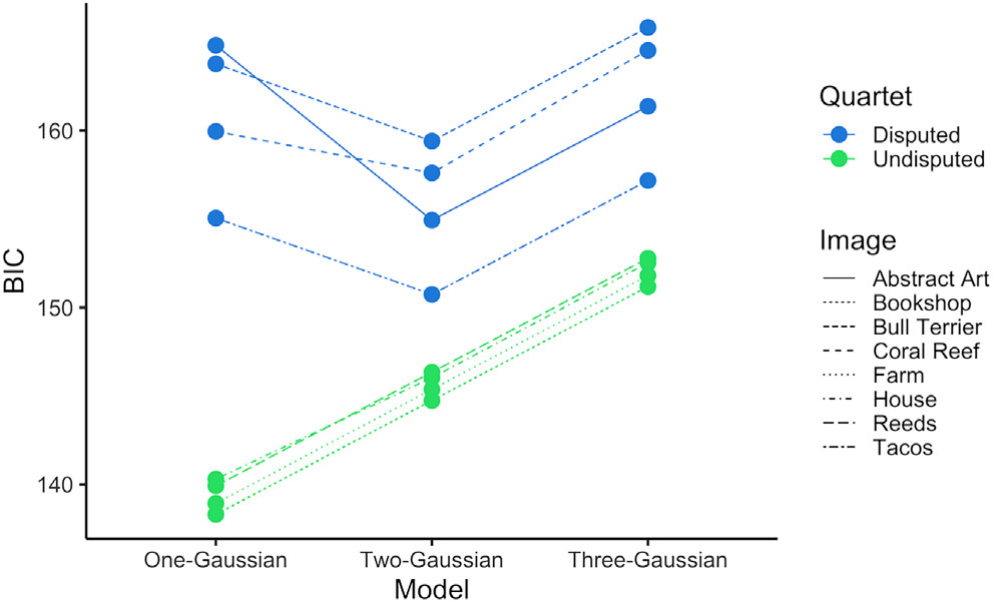
BIC for model fit of all images A lower BIC indicates a better fit. Blue indicates the images in the Disputed-Beauty Quartet and green the Undisputed-Beauty Quartet. Linetype represents the image. Images in the Disputed-Beauty Quartet are better fit by a two-Gaussian model while the images in the Undisputed-Beauty Quartet are better fit by a one-Gaussian model.

We also wanted to test whether the distributions of beauty ratings were better captured by a unimodal, bimodal, or trimodal distribution. We fit three models: a single Gaussian distribution, a mixture of 2 Gaussians, and a mixture of 3 Gaussians (defined in STAR Methods). We calculated their Bayesian Information Criterion (BIC) to assess their fit for the beauty rating distribution of each of the images in the quartets. A lower BIC indicates a better fit. As we anticipated, we found that the images in the Disputed-Beauty Quartet are better fit by our two-Gaussian model while the ones in the Undisputed-Beauty Quartet are better fit by our one-Gaussian model (Figure 4). In most cases, the difference in BIC between the best-fit and second-best models is greater than or equal to 4, which indicates positive evidence in favor of the model with the lowest BIC value.^33^ In the case of the coral reef, the one-Gaussian and two-Gaussian models fit the data equally well.

### Variance estimation

A new group of participants completed three tasks: they rated the beauty of the 8 images in the quartet among 16 other images twice, they estimated the mean and standard deviation of each beauty quartet (*estimated* quartet mean and standard deviation), and they estimated the mean and standard deviation of sets of two, four, or eight numbers. We compare their estimated quartet mean and standard deviation to the actual mean and standard deviation of their beauty ratings. As a control, we also compare their estimated mean and standard deviation for the number sets with the actual values.

**Figure 5:**
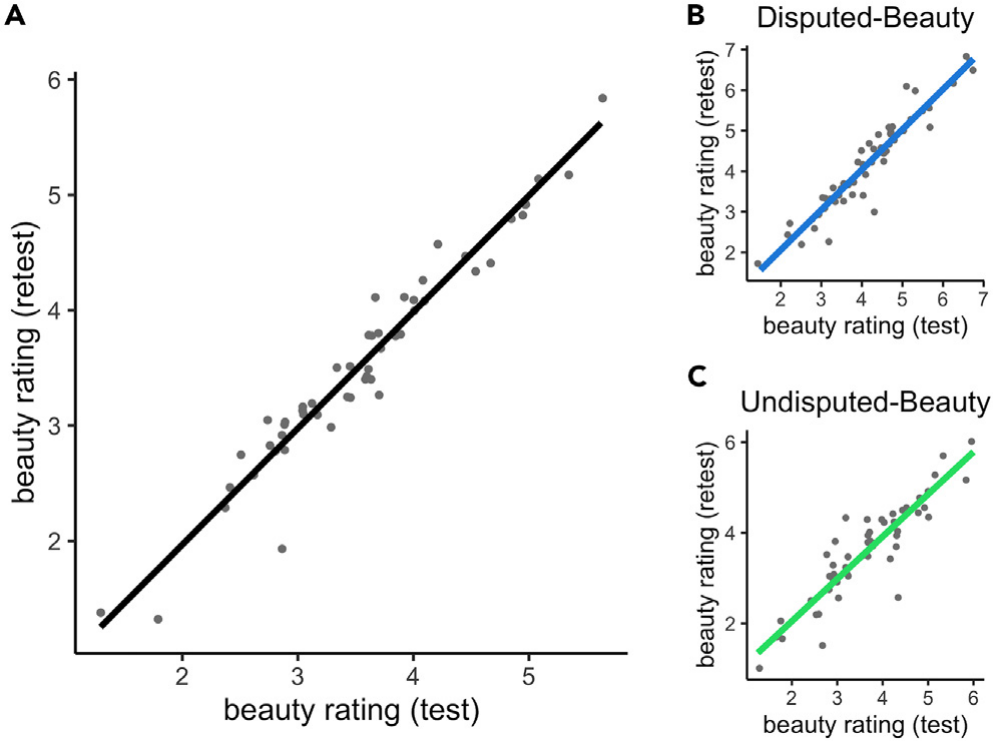
Test-retest correlation of beauty ratings averaged across images for each participant (A) Ratings for the 8 images in the quartet and the 16 foils. (B) Ratings for images in the Disputed-Beauty Quartet and (C) for images in the Undisputed-Beauty Quartet. Points are jittered to prevent overlap. Solid line indicates line of best fit.

A high test-retest correlation between beauty ratings averaged across images for each participant, *r* = 0.93, *p* < 0.001, indicates that participants can reliably rate the beauty of images. This holds after selecting only the images in the Disputed-Beauty Quartet, *r* = 0.95, *p* < 0.001, and the Undisputed-Beauty Quartet, *r =* 0.91, *p* < 0.001. Figure 5 displays the test-retest correlations.

Based on participants’ first beauty ratings, the quartets have the same mean but correspondingly high or low group variance. A one-sided, two-sample paired *t-*test shows no significant difference in mean beauty rating between the quartets, *t*(3) = 1.62, *p* = 0.102, *d* = 0.81. We did observe a significant difference in standard deviation between the quartets, both for raw ratings, *t*(3) = 7.94, *p* < 0.001, *d* = 3.96, and for normalized ratings, *t*(3) = 2.70, *p* = 0.036, *d* = 1.35. Note that even though the degrees of freedom in our statistical analyses are small, in both cases, the mean standard deviation for the Disputed-Beauty Quartet was larger (1.3:1.1 for raw ratings and 1.8:1.5 for normalized ratings). These results replicate those of the image rating task and show that the quartets deliver high and low variance.

**Figure 6:**
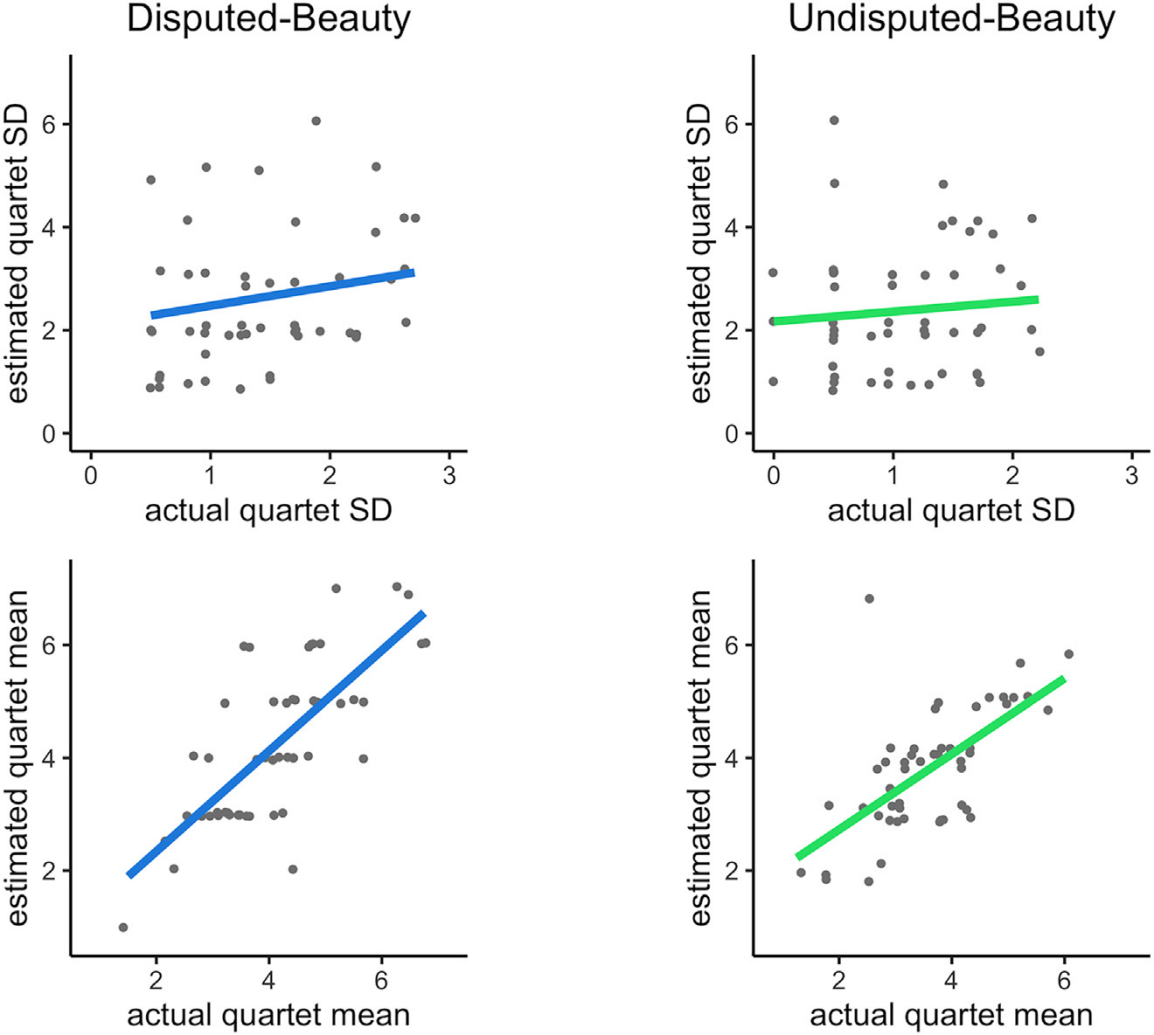
Estimated vs. actual beauty rating standard deviation and mean The left column corresponds to beauty ratings of the Disputed-Beauty Quartet and the right to the Undisputed-Beauty Quartet. Points are jittered to prevent overlap. Solid line indicates line of best fit.

Correlation analyses show that participants can accurately estimate the mean of their beauty ratings but, surprisingly, not the variance. We excluded the data of two participants who reported estimated means of 18 or above since these were extreme outliers. The participants’ *estimated* quartet mean strongly correlates with their actual quartet mean for both the Disputed-Beauty Quartet, *r* = 0.74, *p* < 0.001, and Undisputed-Beauty Quartet, *r* = 0.64, *p* < 0.001. However, there is no significant correlation between participant’s estimated and actual quartet standard deviations for the Disputed-Beauty Quartet, *r* = 0.18, *p* = 0.205, and Undisputed-Beauty Quartet, *r* = 0.09, *p* = 0.515 (Figure 6). At the group level, we do not find any difference in the estimated standard deviation of the quartets, *t*(51) = 1.15, *p* = 0.12, *d* = 0.16. Thus, participants are unable to estimate beauty variance.

**Figure 7:**
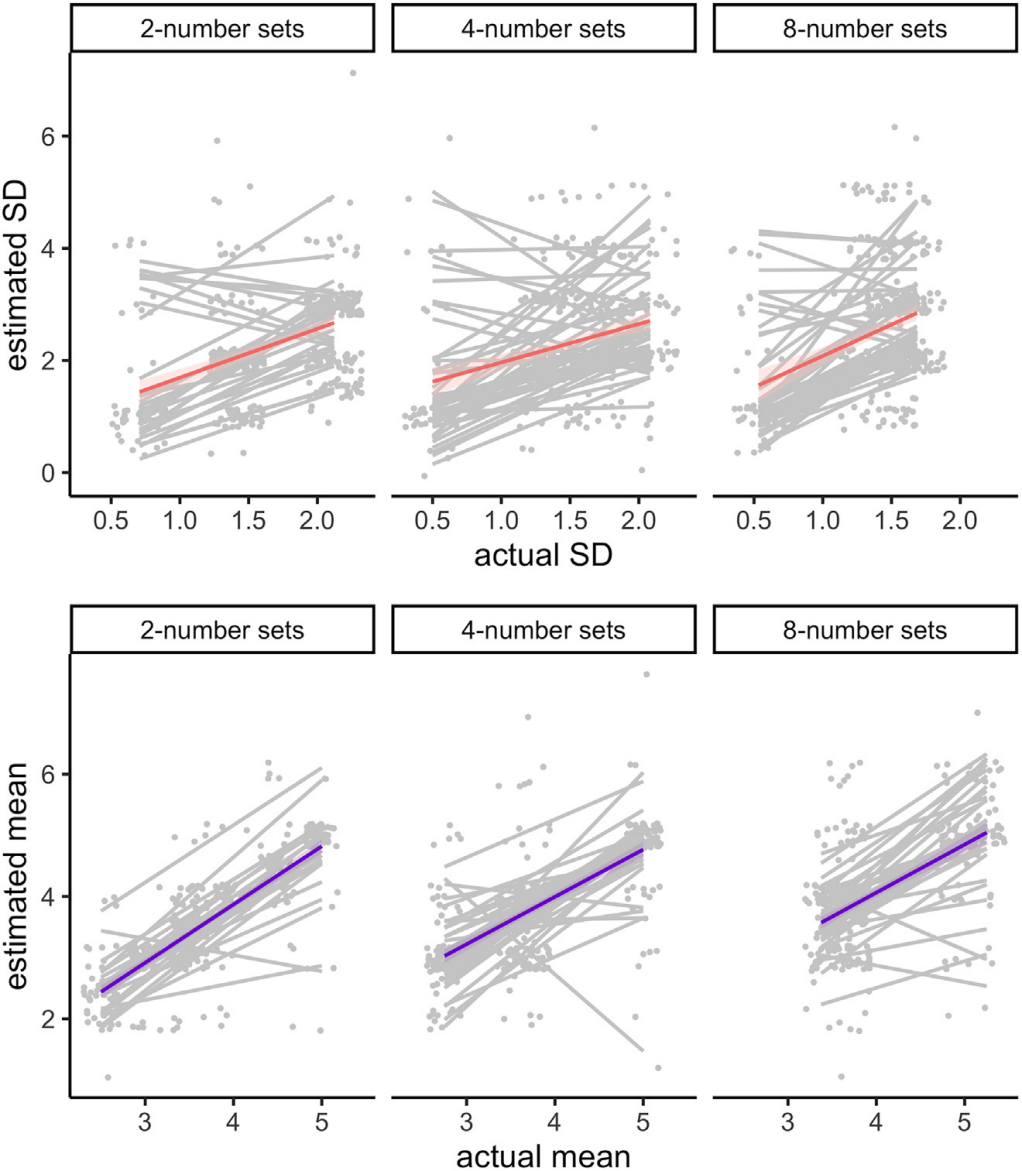
Estimated vs. actual standard deviation and mean of number sets of two, four, or eight numbers Gray lines display the data for each participant and colored lines indicate the group average. Ribbons correspond to standard errors.

We obtained a similar result for the numbers. There are strong correlations between participants’ estimates of the mean of numbers and their actual means for set sizes of two, *r =* 0.76, *p* < 0.001, four, *r =* 0.65, *p* < 0.001, and eight numbers, *r =* 0.56, *p* < 0.001. There are weak yet significant correlations between participants’ estimates of the standard deviation of the numbers and their actual standard deviation for set sizes of two, *r =* 0.41, *p* < 0.001, four, *r =* 0.28, *p* < 0.001 and eight numbers, *r =* 0.33, *p* < 0.001. Figure 7 displays these results.

**Figure 8:**
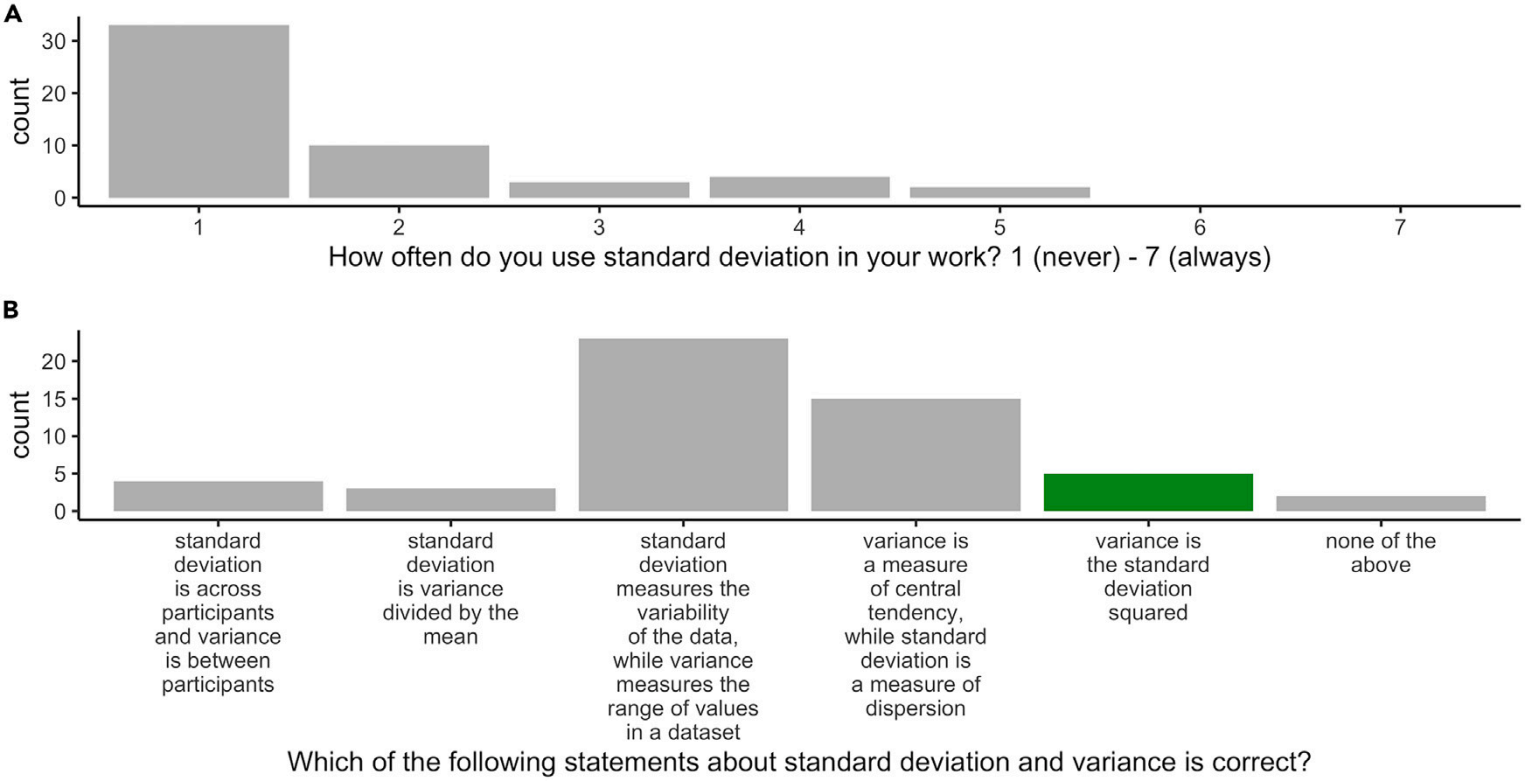
Variance use and knowledge (A) Distribution of answers to questions about variance use and knowledge. In (B), the correct answer is in green.

The observed difficulty to estimate variance is further supported by participants rarely using variance in their work (Figure 8A). Moreover, only 10% of participants were able to accurately identify a correct statement about standard deviation and variance (Figure 8B).

## Discussion

To raise awareness about variance in everyday experiences of beauty, we present two quartets of images with the same across-participant beauty mean: The Disputed-Beauty Quartet comprises four images with high group and quartet variance, and the Undisputed-Beauty Quartet comprises four images with low group and quartet variance. Using these quartets, we show that participants reliably rate the beauty of images and estimate their quartet mean but are unable to estimate their quartet variance. For numbers, we found that participants could estimate variance, but only weakly.

Participant’s difficulty to estimate variance is not unique to beauty ratings. Previous research in mathematics and education has discussed the general population’s unfamiliarity with statistical reasoning. Students struggle with reasoning about variability, and even when students can report and calculate summary statistics, they rarely understand their meaning and importance.^34^^,^^35^^,^^36^ From doctors to lawyers, many struggle with basic probabilistic and statistical thinking.^37^ For decades, scholars have pushed for more emphasis on variance in school math.^38^^,^^39^^,^^40^^,^^41^

Even though beauty variance is invisible, it matters. Our participants were unable to judge beauty variance, but variance in beauty ratings matters practically and theoretically. For beauty in particular, and in general, variance is essential for prediction, explanation, and control.^38^ Any model of beauty prediction must cope with the variation of beauty within and across participants. This is especially relevant for social media and advertisement, which emphasize catering to individual taste.^42^ Recognizing that precision is limited by heterogeneous variance is key to building an effective model of beauty. Theoretically, whether beauty lies in the beholder or is a property of the stimulus is a question of variance. Estimating the two kinds of variance is a step toward reconciling these two possibilities.

Our quartets show that variance in beauty judgment is heterogeneous. This variance may come from many sources, including individual differences in expertise, conformity, or trends.^24^^,^^43^^,^^44^ Of course, it is an oversimplification to suppose that each person rates aesthetics in only one way,^45^ but supposing anything more complicated would add too many degrees of freedom to our model. Regardless of where variance comes from, heterogeneity in variance has methodological implications. Conventional psychological analysis methods emphasize sample means. Typical parametric tests, like *t*-tests and ANOVAs, estimate the significance of mean differences. When the variance is low, all samples will be close to the mean. When the variance is high, most samples will be far from the mean so that the mean by itself is less predictive. Based on our model comparison, images in the Disputed-Beauty Quartet are best summarized by a sum of Gaussians. Given that the distributions encountered in psychophysics are typically unimodal, it seems misleading to summarize a multimodal distribution with just the mean. Linear mixed-effects models pose a great alternative to study the main effects of independent variables while accounting for high group variance.^46^ Such models allow adding random intercept or slope parameters to account for the variance introduced by different participants or stimuli. For example, linear mixed-effects models can be used to effectively quantify idiosyncratic vs. shared contributions to judgment,^47^ estimate sequential effects on aesthetic judgment,^48^ and calculate the contribution of self-relevance to aesthetic ratings of art.^49^ The latter also includes explicit advice on using linear mixed-effects models to partition the variance of aesthetic judgments.

### Limitations of the study

In designing beauty studies, it is important to be aware that there are at least two different ways that participants can understand a request to judge beauty. Participants can rate beauty based on what they believe are beauty standards (e.g., how the art world would rate it). Alternatively, and our focus here, participants can rate beauty based on their own feelings at some moment. To encourage the latter, our study asks participants, “How much beauty do you feel from this image right now?” That’s a useful operationalization of felt beauty, but one could imagine probing to assess how participants understand and reply to the question. Another issue is that our variance estimation task measures only the participant’s ability to predict their own quartet variance, and not group variance. Conceivably, it is harder to estimate group variance than quartet variance. Perhaps the unsettled debate on the universality of beauty stems from scholars implicitly taking positions on the relative importance of the two kinds of variance without measuring them.

### Conclusion

Beauty variance is essential to prediction, both theoretically and practically. We present the Disputed-Beauty and Undisputed-Beauty quartets to show heterogeneity of variance in beauty, both for a participant across stimuli and for a stimulus across participants. The quartets have either high- or low-variance images with high typicality and a given mean beauty. They also have high or low variance across participants (*group* variance) and correspondingly high or low variance across images for each participant (*quartet* variance). We use the quartets to uncover participants’ inability to estimate variance. We hope that our quartets help provoke research questions, statistical analyses, and conclusions that embrace variance heterogeneity.

## STAR★Methods

### Key resources table

**Table:** 

REAGENT or RESOURCE	SOURCE	IDENTIFIER
**Deposited data**		

Raw and analyzed data	This manuscript	https://doi.org/10.17605/OSF.IO/W2EN5

**Software and algorithms**		

Analysis and modeling code	This manuscript	https://doi.org/10.17605/OSF.IO/W2EN5

### Resource availability

#### Lead contact

Further information and requests should be directed to and will be fulfilled by the lead contact, Maria Pombo (maria.pombo@nyu.edu).

#### Materials availability

Links to images are available at: OSF: https://osf.io/w2en5. Qualtrics surveys are available upon request to lead contact.

#### Data and code availability

• All stimuli and raw datasets have been deposited at an OSF repository and are publicly available as of the date of publication. Access link is listed in the key resources table.
• All original experiment and analysis code have been deposited at the same OSF repository and are publicly available as of the date of publication. Access link is listed in the key resources table.
• Any additional information required to reanalyze the data reported in this paper is avialable from the lead contact upon request.

### Experimental model and study participant details

In total, 209 online participants took part in our study, all recruited via Prolific (https://prolific.com). All participants were in the US, spoke English as their first language, and had normal or corrected-to-normal vision. Additional demographics and participant breakdown per task are described below. All participants were adults (ages 18-83) and balanced across sex. 20 of the participants in the image crowdsourcing task were art students. In our image crowdsourcing task, we included art students using a filter on Prolific as a way to increase the number of art pieces in our image set. We did not collect any ancestry, race, or ethnicity data. All participants gave informed consent in accordance with the Declaration of Helsinki. This experiment was approved by the New York University Committee on Activities Involving Human Subjects (IRB-FY2019-2456).

For the Disputed-Beauty Quartet, the Image Crowdsourcing sample had 69 participants (33 females, 35 males, 1 preferred not to say; ages 19-77, *M* = 31.38 ± 11.65 years) and the Image Rating sample consisted of 25 participants (18 females, 7 males; ages 18-83, *M =* 36.04 ± 15.26 years). For the Undisputed-Beauty Quartet, the Image Crowdsourcing sample included 40 participants (17 females, 23 males; ages 22-67, *M =* 37.38 ± 10.50 years) and the Image Rating task sample consisted of 25 participants (11 females, 14 males; ages 20-64, *M =* 35.22 ± 12.89 years). 50 participants completed the Variance Estimation task (24 females, 26 males; ages 22-72, *M =* 39.98 ± 12.04).

Participants were randomly allocated to experimental conditions, and no participant completed more than one session. For the image crowdsourcing task we recruited enough participants to collect 180 images in each condition. The image rating task and variance estimation task both had sample sizes of 50, which are consistent with previous work done in the lab.^48^

### Method details

#### Image Crowdsourcing

After giving consent and answering demographics questions, 109 participants completed one of two simple image crowdsourcing tasks, one for disputed and one for undisputed beauty. For the disputed beauty task, the instructions were the following: “Anything can be beautiful. Please copy the link to three different images that, in your experience, represent something you would encounter typically that is disputed in terms of its beauty.” For the undisputed beauty task, the instructions were the following: “Beauty can be controversial, but we are looking for instances of agreement. Please upload below three different photos that, in your experience, represent something you would encounter typically that everyone would consider is “meh” (5 out of 10) in terms of its beauty.” After having copyright issues with some images in the disputed beauty task, we asked participants assigned to the undisputed beauty task to submit pictures that they had taken with their phones and asked them to sign a photo release. To motivate them to think critically in this challenging task, we offered a $100 bonus if their images were selected as one of the final four images, and we offered an explanation of what we meant by “meh” undisputed beauty: “If you need help, think of something that is very beautiful (e.g., a sunset) and something that is very ugly (e.g., a cockroach). Now think of something that would fall right in the middle of those in terms of its beauty. Do you think everyone would agree with you?”

These and all other tasks were programmed using Qualtrics (https://www.qualtrics.com/). The task lasted 2.5 minutes on average, and participants were compensated minimum wage in New York City for their contribution ($15/hour).

We then examined each link and downloaded the corresponding image. We excluded links that were broken or were ambiguous on the target image (e.g., we excluded links to websites that had multiple diverse images). We also excluded images that displayed violent content and converted all images in .webp format to .jpeg. When the linked image had very low resolution, we searched for similar images through Unsplash (https://unsplash.com/), an open-source, high-resolution, image database.

After adding image suggestions from colleagues and friends, we ended up with 182 disputed beauty images and 180 undisputed beauty images.

#### Image Rating

After giving consent and answering demographics questions, 50 new participants saw either the 182 disputed beauty images or the 180 undisputed images, one by one, and were asked to rate their beauty and their typicality in two different Likert scales of radio buttons displayed below the image. They rated, on a scale from 1 (not at all) to 7 (very much), how much beauty they felt from the image and rated, on a scale from 1 (not at all) to 7 (very), how typical the image was. Images were presented in their original aspect ratio. We fixed the horizontal axis at 400px, which, on a 2880px by 1800px display, corresponds to about 5.3° of visual angle for an observer at a 50 cm distance from the screen. We did not control viewing distance but 50 cm is typical. We instructed participants to use a desktop computer to complete the task. 37 participants used Chrome, 7 Firefox, 2 Safari, 3 Edge, and 1 Opera. Their screen resolutions ranged from 1222x688 to 2560x1440 pixels. It took participants approximately 33 minutes to complete this survey.

#### Variance Estimation

50 new participants completed an 18-minute survey. Participants first rated the beauty of the 8 images in the quartets among 16 foil images, presented one at a time in their original aspect ratio. We fixed the horizontal axis at 400px, which, on a 2880px by 1800px display, corresponds to about 5.3° of visual angle for an observer at a 50 cm distance from the screen. We did not control viewing distance but 50 cm is typical. Half of the foil images came from the set of 182 disputed-beauty images and half from the 180 undisputed-beauty images. Participants then rated the beauty of the 24 images again. We did this as a measure of participant test-retest reliability.

Participants then completed a variance estimation task. We showed participants each quartet, in a counterbalanced order and in the same layout as Figures 1 and 2, and asked them the following: “We are interested in your estimate of the average and dispersion of the beauty of each image above. AVERAGE: Mean is the average rating, or the sum of all ratings divided by the number of ratings. The mean of 1, 3, and 5 is 3. DISPERSION: Standard deviation is a measure of the amount of variation or dispersion of a set of values. A low standard deviation indicates that the values tend to be close to the mean of the set, while a high standard deviation indicates that the values are spread out over a wider range. The dispersion of 1, 3, 5 is 2. On a scale from 1 (none at all) to 7 (very much), think about the beauty you feel from each of these images above. Off the top of your head, what is your estimate of their average? Off the top of your head, what is your estimate of their dispersion?” Participants were allowed to input any number, including fractions.

Lastly, as a control, participants completed a similar task where instead of estimating the average and dispersion of images, they did so for numbers. We showed participants eight sets of two, four, and eight numbers between 1 and 7. Examples of the number sets are available at OSF: https://osf.io/w2en5/. The instructions were the following: “The numbers above are at least 1 and at most 7. We are interested in your estimate of their average and dispersion.” We also included the same definitions for average and dispersion described above and asked the same open-ended questions. The order of presentation of the number sets was randomized.

We included an attention check where participants had to identify which of four images they had seen in the survey. We also asked participants to rate, on a scale from 1 (never) to 7 (often), how often they use standard deviation in their work. Finally, participants had to select which of seven statements was true about standard deviation and variance. The correct statement was “variance is standard deviation squared.” We used chatGPT to generate plausible but incorrect statements about standard deviation and variance as alternatives (Figure 8).

### Quantification and statistical analysis

#### Image Rating

After data collection, we calculated the mean and standard deviation of both the beauty and typicality ratings. We selected two subsets of four images. The Disputed-Beauty Quartet has middle-range mean beauty ratings, *high* beauty standard deviation, and high typicality. The Undisputed-Beauty Quartet has middle-range mean beauty ratings, *low* beauty standard deviation, and high typicality. We considered two images with a mean beauty rating difference of 0.5 or less to have similar mean beauty ratings. All our images have mean beauty ratings between 3.5 and 4. We considered images with typicality scores of 3 or above.

To select images with high standard deviation, we considered two types of variance: quartet and group variance. *Quartet variance* refers to the standard deviation across images in the quartet for one participant. *Group variance* refers to the standard deviation of beauty ratings per image across participants. We conducted a brute-force search where we computed all combinations of four images in each of our image sets with mean beauty ratings between 3.5 and 4 and mean typicality ratings of 3 or above. For each combination of four images, we calculated the mean across participants of the difference between the highest and lowest beauty ratings. Among the sets of four images with a mean difference in the top 25%, we selected a set of four that also had high group variance. We considered several factors in selecting the final quartets such as excluding images that had faces or identifiable information, reviewing image copyright, and aiming to select quartets for which the images covered diverse categories (e.g., only one painting, only one dog). We defined categories broadly and aimed to select images that covered different aesthetic contexts such as nature, art, and everyday scenes. To select images with low standard deviation, we followed the same procedure except we looked at the sets of four images with a mean difference in the bottom 25% for images with low group variance. As a sanity check, we conducted two one-sided two-sample *t*-tests to ensure that the mean beauty did not differ significantly across the quartets but the standard deviation did.

We also tested whether the beauty distributions were best represented by one, two, or three Gaussian distributions. We defined a model which is a mixture of three Gaussians:

(Equation 1) $$f\left(x\right)=\frac{1}{\sigma \sqrt{2\pi }}\;e^{\frac{-1}{2}{\left(\frac{x-\mu }{\sigma }\right)}^{2}}$$

where the mean μ is a free parameter. In order to constrain the search space to reasonable solutions, the standard deviation σ is constrained to 1.43, which is the average standard deviation of the beauty distributions for the Undisputed-Beauty Quartet. Assuming that counts are independent with a Poisson distribution, we define the likelihood function as:

(Equation 2) $$L\left(\mu ,\sigma ,⋋\right)=\prod_{i=1}^{N}\frac{e^{-⋋}}{y_{i}!}{⋋}^{y_{i}}$$

where N is the number of data points, $x_{i}$ is the *i*th data point, $y_{i}$ is the value of the *i*th data point, and

(Equation 3) $$⋋=A_{1}f_{1}\left(x_{i}\right)+A_{2}f_{2}\left(x_{i}\right)+A_{3}f_{3}\left(x_{i}\right)$$

and

(Equation 4) $$1=A_{1}+A_{2}+A_{3}$$

We considered three versions of this model. In the one-Gaussian model, $A_{2}=A_{3}=0$. In the two-Gaussian model, $A_{3}=0$. Note that Equation 4 reduces the number of degrees of freedom when more than one of the A’s is nonzero. In the end, our first model has one free parametert, our second model has three, and our third has five.

We use the *optim()* function in R^50^ to find the values of the free parameters that minimize the negative log of our likelihood function (Equation 2). We use the “L-BFGS-B” method,^51^ which allows us to constrain the free parameters. μ is constrained to values in our Likert scale (between 1 and 7). When they are nonzero, $A_{1}$, $A_{2}$, and $A_{3}$ are constrained to values between 0 and 1.

We fit these models individually to the 8 images in our quartets. To assess their fit, we calculate their Bayesian Information Criterion (BIC), which takes into account the minimum negative log-likelihood as well as the number of free parameters. A lower BIC indicates a better model fit.

#### Variance Estimation

From each participant, we obtained two beauty ratings for each image, their estimated mean and standard deviation for each beauty quartet (*estimated* quartet mean and variance), and their estimated mean and standard deviation for each number set. Based on their first beauty ratings, we also calculated the actual mean and standard deviation of their beauty ratings of each quartet (*actual* quartet mean and variance). Lastly, we calculated the actual mean and standard deviation of the number sets.

To measure beauty rating reliability, we first calculated the test-retest Pearson’s correlation in beauty ratings across participants. To test how well participants could estimate the dispersion of their own ratings, we computed a Pearson’s correlation between the estimated quartet standard deviation and the actual quartet standard deviation. We did the same for the estimated quartet mean vs. actual quartet mean and for the estimated vs. actual mean and standard deviation of the number sets. We used a one-sided, two-sample paired *t*-test to assess the difference in estimated standard deviations between the quartets. Lastly, we calculated the difference in group variance across the two quartets in two ways. First, for each quartet, we calculated the standard deviation of the beauty ratings for each image across participants and performed a one-sided, two-sample *t*-test between the two quartets. Second, for each quartet, we took each participant’s rating for the four images and subtracted their mean. We refer to this as the “normalized” ratings. We then calculated the standard deviation of the normalized ratings for each image and performed a one-tailed two-sample *t*-test between the two quartets. Ultimately, our analyses allowed us to test how reliably participants rate beauty and how well they can estimate the mean and standard deviation of beauty and numbers. In our statistical analyses, we assume that there are no outliers, that the data are normally distributed, and that variance is homogeneous. For all *t*-tests, we calculated the effect size with Cohen’s *d*.

All data analysis was performed using R Studio (R version 4.2.2) and all code is available here: https://osf.io/w2en5/.

## References

[1] Nadal M., Ureña E., Nadal M., Vartanian O. (2022). The Oxford Handbook of Empirical Aesthetics.

[2] Babbitt M., Woods M., Washburn M.F. (1915). Affective Sensitiveness to Colors, Tone Intervals, and Articulate Sounds. Am. J. Psychol..

[3] Clark H., Quackenbush N., Washburn M.F. (1913). A Suggested Coefficient of Affective Sensitiveness. Am. J. Psychol..

[4] Chandler A.R. (1934).

[5] Berlyne D.E. (1971). Aesthetics and Psychobiology. J. Aesthet. Art Critic..

[6] Brielmann A.A. (2021). Aesthetics, Empirical | Internet Encyclopedia of Philosophy. https://iep.utm.edu/emp-aest/.

[7] Bertamini M., Rampone G., Nadal M., Vartanian O. (2020). The Oxford Handbook of Empirical Aesthetics.

[8] Makin A.D.J., Pecchinenda A., Bertamini M. (2012). Implicit affective evaluation of visual symmetry. Emotion.

[9] Höfel L., Jacobsen T. (2003). Temporal Stability and Consistency of Aesthetic Judgments of Beauty of Formal Graphic Patterns. Percept. Mot. Skills.

[10] Makin A.D., Helmy M., Bertamini M. (2018). Visual cortex activation predicts visual preference: Evidence from Britain and Egypt. Q. J. Exp. Psychol..

[11] Chuquichambi E.G., Vartanian O., Skov M., Corradi G.B., Nadal M., Silvia P.J., Munar E. (2022). How universal is preference for visual curvature? A systematic review and meta-analysis. Ann. N. Y. Acad. Sci..

[12] Corradi G., Munar E., Nadal M., Vartanian O. (2019). The Oxford Handbook of Empirical Aesthetics.

[13] Palmer S.E., Schloss K.B., Sammartino J. (2013). Visual Aesthetics and Human Preference. Annu. Rev. Psychol..

[14] Brielmann A.A., Pelli D.G. (2019). Intense Beauty Requires Intense Pleasure. Front. Psychol..

[15] Hönekopp J. (2006). Once more: is beauty in the eye of the beholder? Relative contributions of private and shared taste to judgments of facial attractiveness. J. Exp. Psychol. Hum. Percept. Perform..

[16] Jacobsen T. (2004). Individual and group modelling of aesthetic judgment strategies. Br. J. Psychol..

[17] Leder H., Goller J., Rigotti T., Forster M. (2016). Private and Shared Taste in Art and Face Appreciation. Front. Hum. Neurosci..

[18] Vessel E.A., Maurer N., Denker A.H., Starr G.G. (2018). Stronger shared taste for natural aesthetic domains than for artifacts of human culture. Cognition.

[19] Vessel E.A., Rubin N. (2010). Beauty and the beholder: Highly individual taste for abstract, but not real-world images. J. Vis..

[20] Wallisch P., Whritner J.A. (2017). Strikingly Low Agreement in the Appraisal of Motion Pictures. Projections.

[21] Aleem H., Correa-Herran I., Grzywacz N.M. (2020). A Theoretical Framework for How We Learn Aesthetic Values. Front. Hum. Neurosci..

[22] Street N., Forsythe A.M., Reilly R., Taylor R., Helmy M.S. (2016). A Complex Story: Universal Preference vs. Individual Differences Shaping Aesthetic Response to Fractals Patterns. Front. Hum. Neurosci..

[23] Whitfield A. (1984). Individual Differences in Evaluation of Architectural Colour: Categorization Effects. Percept. Mot. Skills.

[24] Leder H., Tinio P.P.L., Brieber D., Kröner T., Jacobsen T., Rosenberg R. (2019). Symmetry Is Not a Universal Law of Beauty. Empir. Stud. Arts.

[25] Myers C., Wallisch P. (2020).

[26] Corradi G., Chuquichambi E.G., Barrada J.R., Clemente A., Nadal M. (2020). A new conception of visual aesthetic sensitivity. Br. J. Psychol..

[27] Clemente A., Pearce M.T., Nadal M. (2022). Musical aesthetic sensitivity. Psychol. Aesthet. Creativ. Arts.

[28] Mas-Herrero E., Marco-Pallares J., Lorenzo-Seva U., Zatorre R.J., Rodriguez-Fornells A. (2013). Individual Differences in Music Reward Experiences. Music Percept..

[29] Schlotz W., Wallot S., Omigie D., Masucci M.D., Hoelzmann S.C., Vessel E.A. (2021). The Aesthetic Responsiveness Assessment (AReA): A screening tool to assess individual differences in responsiveness to art in English and German. Psychol. Aesthet. Creativ. Arts.

[30] Chen Y.-C., Chang A., Rosenberg M.D., Feng D., Scholl B.J., Trainor L.J. (2022). “Taste typicality” is a foundational and multi-modal dimension of ordinary aesthetic experience. Curr. Biol..

[31] Bignardi G., Smit D.J.A., Vessel E.A., Trupp M.D., Ticini L.F., Fisher S.E., Polderman T.J.C. (2024). Genetic effects on variability in visual aesthetic evaluations are partially shared across visual domains. Commun. Biol..

[32] Aleem H., Grzywacz N.M. (2023). The temporal instability of aesthetic preferences. Psychol. Aesthet. Creativ. Arts.

[33] Raftery A.E. (1995). Bayesian Model Selection in Social Research. Socio. Methodol..

[34] Delmas R., Liu Y. (2005). Exploring students’ conceptions of the standard deviation. Stat. Educ. Res. J..

[35] Garfield J., Ben-Zvi D. (2007). How Students Learn Statistics Revisited: A Current Review of Research on Teaching and Learning Statistics. Int. Stat. Rev..

[36] Mathews D., Clark J.M. (2003).

[37] Sriraman B., Chernoff E.J., Lerman S. (2020). Encyclopedia of Mathematics Education.

[38] Reading C., Shaughnessy J.M., Ben-Zvi D., Garfield J. (2004). The Challenge of Developing Statistical Literacy, Reasoning and Thinking.

[39] Innabi H., Marton F., Emanuelsson J., Burrill G.F., de Oliveria Souza L., Reston E. (2023). Research on Reasoning with Data and Statistical Thinking: International Perspectives Advances in Mathematics Education.

[40] Lehrer R., English L., Ben-Zvi D., Makar K., Garfield J. (2018). International Handbook of Research in Statistics Education Springer International Handbooks of Education.

[41] Pratt D., Kazak S., Ben-Zvi D., Makar K., Garfield J. (2018). International Handbook of Research in Statistics Education Springer International Handbooks of Education.

[42] Pombo M., Pelli D.G. (2022). Aesthetics: It’s beautiful to me. Curr. Biol..

[43] Carbon C.-C. (2010). The cycle of preference: Long-term dynamics of aesthetic appreciation. Acta Psychol..

[44] Mather K.B., Aleem H., Rhee Y., Grzywacz N.M. (2023). Social groups and polarization of aesthetic values from symmetry and complexity. Sci. Rep..

[45] Muth C., Carbon C.-C. (2016). SeIns: Semantic Instability in Art. Art Percept..

[46] Kliegl R., Wei P., Dambacher M., Yan M., Zhou X. (2010). Experimental Effects and Individual Differences in Linear Mixed Models: Estimating the Relationship between Spatial, Object, and Attraction Effects in Visual Attention. Front. Psychol..

[47] Martinez J.E., Funk F., Todorov A. (2020). Quantifying idiosyncratic and shared contributions to judgment. Behav. Res. Methods.

[48] Pombo M., Brielmann A.A., Pelli D.G. (2023). The intrinsic variance of beauty judgment. Atten. Percept. Psychophys..

[49] Vessel E.A., Pasqualette L., Uran C., Koldehoff S., Bignardi G., Vinck M. (2023). Self-Relevance Predicts the Aesthetic Appeal of Real and Synthetic Artworks Generated via Neural Style Transfer. Psychol. Sci..

[50] R Core Team (2013).

[51] Byrd R.H., Lu P., Nocedal J., Zhu C. (1995). A Limited Memory Algorithm for Bound Constrained Optimization. SIAM J. Sci. Comput..

